# Development of a repeat-exposure penile SHIV infection model in macaques to evaluate biomedical preventions against HIV

**DOI:** 10.1371/journal.pone.0194837

**Published:** 2018-03-27

**Authors:** David A. Garber, James Mitchell, Debra Adams, Patricia Guenthner, Frank Deyounks, Shanon Ellis, Kristen Kelley, Ryan Johnson, Charles Dobard, Walid Heneine, Janet McNicholl

**Affiliations:** Laboratory Branch, Division of HIV/AIDS Prevention, Centers for Disease Control and Prevention, Atlanta, Georgia, United States of America; University of Pittsburgh, UNITED STATES

## Abstract

Penile acquisition of HIV infection contributes substantially to the global epidemic. Our goal was to establish a preclinical macaque model of penile HIV infection for evaluating the efficacy of new HIV prevention modalities. Rhesus macaques were challenged once or twice weekly with consistent doses of SHIV_sf_162P3 (a chimeric simian-human immunodeficiency virus containing HIV *env*) ranging from 4–600 TCID_50_ (50% tissue culture infective dose), via two penile routes, until systemic SHIV infection was confirmed. One route exposed the inner foreskin, glans and urethral os to virus following deposition into the prepuce (foreskin) pouch. The second route introduced the virus non-traumatically into the distal urethra only. Single-route challenges resulted in dose-dependent rates of SHIV acquisition informing selection of optimal SHIV dosing. Concurrent SHIV challenges via the prepuce pouch (200 TCID_50_) and urethra (16 TCID_50_) resulted in infection of 100% (10/10) animals following a median of 2.5 virus exposures (range, 1–12). We describe the first rhesus macaque repeat-exposure SHIV challenge model of penile HIV acquisition. Utilization of the model should further our understanding of penile HIV infection and facilitate the development of new HIV prevention strategies for men.

## Introduction

Acquisition of HIV infection via the penis contributes substantially to the global epidemic as approximately one-half of the estimated 35 million adults living with HIV globally are men, a majority of whom acquired HIV through heterosexual transmission [1]. Despite the utility of HIV prevention strategies including condom usage, behavioral counseling, medical circumcision and now pre-exposure prophylaxis (PrEP) with antiretroviral drugs emtricitabine and tenofovir disoproxil fumarate (Truvada^®^), there remains a pressing need to develop additional penis-specific HIV prevention strategies. These could include additional antiretroviral drugs as PrEP, microbicides or vaccines. Nonhuman primate models of rectal or vaginal HIV acquisition are used commonly to assess the protective efficacy of PrEP or HIV vaccines and to facilitate their advancement into clinical studies [2–4]. Such models are often predicated upon performing repeated virus challenges, via the route of interest, using limited doses of simian immunodeficiency virus (SIV) or simian-human immunodeficiency virus (SHIV), such that infections occur within a defined range of virus exposures [5–7]. The repeated nature of the challenges gives great statistical power [7]. With this design, group sizes of as few as six animals in treatment and control arms can demonstrate prevention efficacy as shown in macaque studies of Truvada^®^ as PrEP for rectal or vaginal SHIV acquisition [3]. Maximizing the sensitivity of a repeat-challenge model for discerning protective efficacy of candidate interventions requires that a challenge virus dose be selected that is less than that which uniformly infects all negative control animals following a single exposure, yet is sufficiently high to infect all controls within a practical range of challenges.

The development of repeat-exposure challenge models of penile HIV infection in nonhuman primates has lagged those focused on rectal or vaginal infection routes. This has resulted in advancing candidate HIV interventions for heterosexual men into clinical studies without any preclinical penile efficacy assessments in macaques. Previous work demonstrated the feasibility of infecting rhesus macaques with SIV (strains SIVmac251 or SIVsmE660) via the foreskin/glans following inoculation into the prepuce pouch, immersion of the flaccid penis into the virus inoculum, or instillation of virus into the urethra [8–10]. None of these studies has compared each of these routes in parallel with the same virus, so the extent to which various penile compartments contribute to virus acquisition is not known. A recent report describing early events following penile inoculation of macaques with SIVmac251 showed that many different anatomic areas of the penis including the glans, foreskin and coronal sulcus can be sites of SIV acquisition [11]. In human penile explant studies, the urethra is a susceptible site for HIV—potentially being more susceptible than the glans [12].

Prior macaque penile challenge studies all used SIV (SIVmac251 or SIVsmE660) as the challenge virus, used relatively high doses, sometimes with escalating amounts and sometimes challenging more than once per day [10]. A disadvantage of SIV-based models, as compared with those using recombinant SHIVs containing HIV-1 *env*, is that they are not ideal for evaluating HIV vaccines as prevention, where Env-directed antibody or T cell responses may be critical components of vaccine efficacy. Furthermore, SIVsmE660 infection of rhesus macaques is dependent on host TRIM5 genotypes [8], whereas SIVmac251 or SIVmac239-based SHIVs, including SHIV_sf_162P3, are not [10, 13–15]. Thus, recombinant SHIVs are widely used in macaque HIV vaccine evaluation studies, as well as in the testing of other HIV preventions, including PrEP. Despite the importance of SHIVs for HIV prevention research, there is no penile macaque SHIV infection model.

Here, we aimed at developing a repeat SHIV challenge model, with well-defined SHIV exposures to penile tissues of both the inner foreskin, glans and the urethra—sites which, in humans, all may be exposed to vaginal or rectal secretions during insertive intercourse as potential portals of HIV entry. Our strategy was to evaluate, in parallel, non-traumatic single-tissue virus titrations using repeated exposures, to develop a macaque model of penile SHIV acquisition analogous to the repeat exposure models used successfully to model rectal or vaginal HIV acquisition [3]. The ultimate goal was to foster preclinical evaluation and advancement of PrEP, HIV vaccines and other HIV prevention strategies for men. We used SHIVsf162P3, a well-characterized virus containing an SIVmac239 backbone that expresses HIV-1 clade B proteins including Tat, Rev, Vpu and Env, which is CCR5-tropic and belongs to the tier-2 neutralization category [16]. By using a recombinant SHIV containing HIV-1 *env*, the model has greater applicability for evaluating a range of preventions, from microbicides to HIV vaccines. Because this virus has been the primary strain used in our rectal and vaginal prevention studies in macaques [3], using it for penile acquisition and prevention studies would allow us to compare such studies across three different mucosal compartments.

## Materials and methods

### Ethics statement

Twenty-one adult male Indian-origin rhesus macaques (*Macaca mulatta*) were used in this study. Animals averaged 7.3 years of age (range = 4.8–10.7 years) and were housed at the Centers for Disease Control and Prevention (CDC; Atlanta, GA) in accordance with the Guide for the Care and Use of Laboratory Animals (8^th^ edition) in an AALAC-accredited facility, according to institutional standard operating procedures. For housing, macaques were maintained in cages that met or exceeded the minimum size requirements as stipulated in the Guide (cage dimensions of 30 inches length x 30 inches width x 30 inches height). Animals were provided enrichments that included objects to manipulate, assortments of fresh food selections (fruits, vegetables), suitable feeding methods (foraging and task-oriented), and humane interactions with caregivers and research staff. Prior to the initiation of virus challenges, compatible macaques were pair-housed to the extent possible. Animal studies were approved by the CDC Institutional Animal Care and Use Committee (IACUC, protocols 2567GARMONC, 2726DOBMONC). To minimize animal discomfort or suffering, all biomedical procedures were performed on animals under ketamine (10mg/kg) or Telazol (2–6mg/kg) anesthesia.

### Virus stock

The cell-free challenge virus stock used in this study was derived from CCR5-utilizing SHIV_sf_162P3, initially obtained from the NIH AIDS Reagent Repository (SHIV162P3-NIH-2 (2007)). Briefly, SHIV_sf_162P3 was amplified in cynomolgus macaque (*Macaca fascicularis*) peripheral blood mononuclear cells (PBMCs) following in vitro depletion of CD8+ cells (Dynabeads CD8, ThermoFisher) and stimulation with Concanavalin-A (Sigma-Aldrich), and used to infect a male cynomolgus macaque via the prepuce pouch. Virus cultured from PBMCs obtained from the infected animal at 13 weeks following infection was further expanded in rhesus macaque PBMCs (CD8-depleted/ConA-stimulated, as above) and clarified via centrifugation. The undiluted challenge virus stock contains 6.8E09 viral RNA copies/ml and has a titer of 2,430 TCID_50_/ml, determined on whole, unstimulated primary rhesus PBMCs.

### Animal procedures

**Fig 1:**
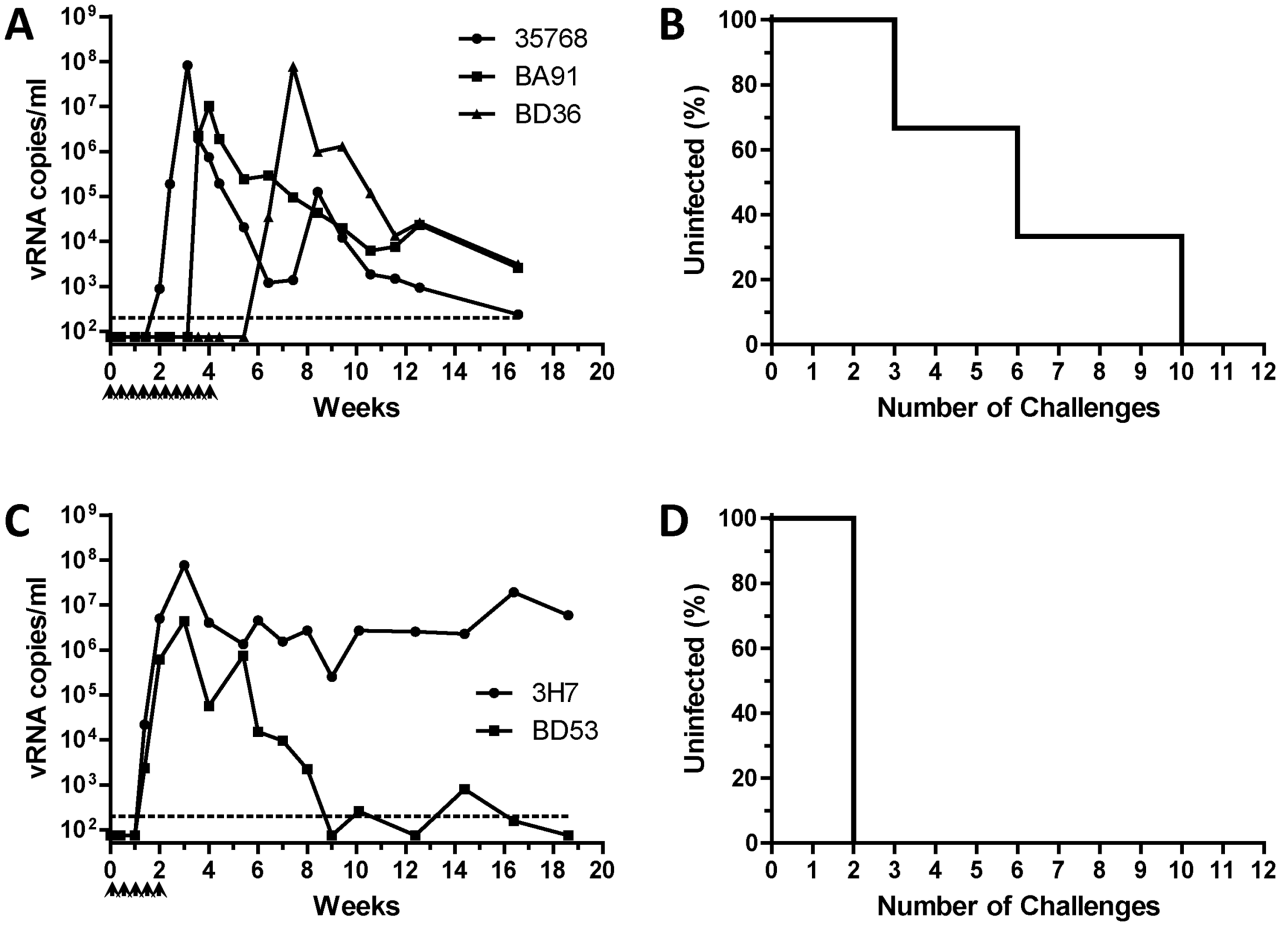
Infection of rhesus macaques following repeated SHIV challenges via the prepuce pouch. Beginning at week 0, rhesus macaques were challenged twice weekly with 200 TCID_50_ (A, B) or 600 TCID_50_ (C, D) SHIV via the prepuce pouch with until systemically infected, as evidenced by detectable plasma viremia via RT-qPCR viral load assay. Viral RNA copies per ml plasma are shown as a function of study week (A, C). Percentages of animals remaining uninfected at seven days following administration of the indicated challenge number are shown (B, D). Dotted lines indicate the limit of quantitation of viral load assay (200 copies/ml). Identification codes represent individual animals. Arrowheads denote SHIV challenges.

**Fig 2:**
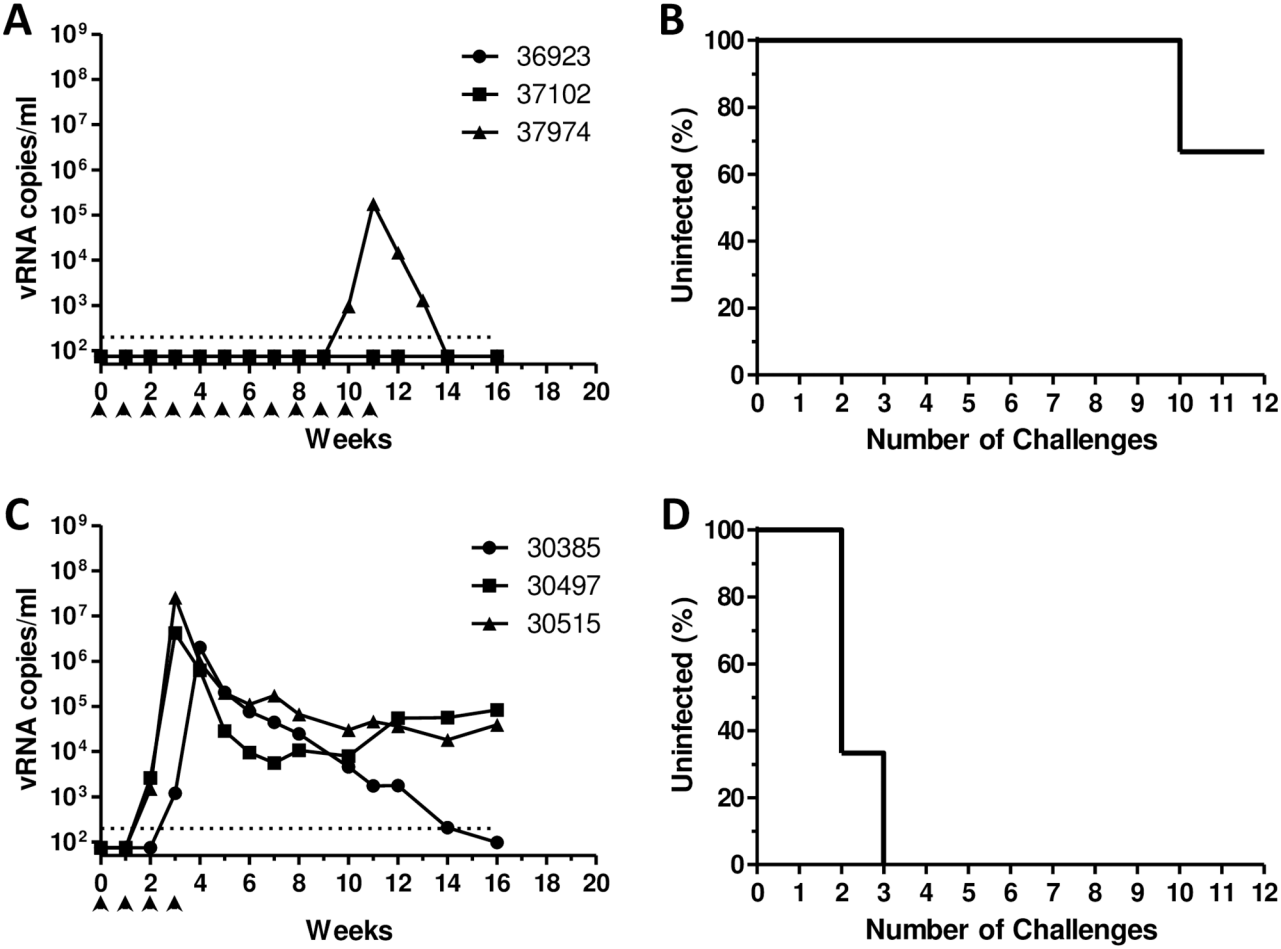
Infection of rhesus macaques following repeated SHIV challenges via the urethra. Beginning at week 0, rhesus macaques were challenged once weekly with 4 TCID_50_ (A, B) or 16 TCID_50_ (C, D) SHIV via the urethra using the ‘no contact’ technique, as described, until systemically infected (detectable plasma viremia) or until having received 12 challenges maximum. Viral RNA copies per ml plasma are shown as a function of study week (A, C). Percentages of animals remaining uninfected at seven days following administration of the indicated challenge number are shown (B, D). Dotted lines indicate the limit of quantitation of viral load assay (200 copies/ml). Identification codes represent individual animals. Arrowheads denote SHIV challenges.

Rhesus macaques were challenged with 4–600 TCID_50_ SHIV, once or twice weekly, via the prepuce pouch and/or urethra, as indicated. Prepuce pouch inoculations were performed as described in Yeh, *et al* [8] with minor modifications including reducing the inoculum volume by 50% and using SHIV_sf_162P3 instead of SIVsmE660. Briefly, blunt forceps were used to gently extend the foreskin to create a pouch into which 0.25ml of virus was dripped, thereby exposing the inner foreskin, glans and urethral os to the virus inoculum. For studies presented in Fig 1, the foreskin was then held opposed for 30 minutes using a Schwartz micro serrefine (Fine Science Tools, Inc.) as a precaution against leakage of the virus inoculum. However, using a serrefine in this capacity was found unnecessary, and the procedure was not used during the dual-route challenge study. Urethral inoculations were performed by pairs of trained animal technicians using a ‘no contact’ technique, in which the urethral epithelium was exposed only to the liquid virus inoculum, to preclude inadvertent abrasion of the urethral epithelium by the inoculation procedure itself. This is in contrast to a previous macaque urethral inoculation study where 1 ml of cell-free SIV was inserted 1 cm into the urethra using a pediatric nasogastric feeding tube [9]. First, the lobes of the glans penis were manually flared to expose the navicular fossa. A flexible sterile filter tip (Fisherbrand Cat#02-707-169), fitted on a micropipettor, was then positioned 1–5mm above the center of the exposed urethral opening at the proximal end of the navicular fossa. While avoiding all direct contact of the pipet tip with the epithelia, a 15 or 20 microliter volume of virus inoculum was expelled as a drop from the end of the pipet tip into the urethral opening in the studies described in Figs 2A and 2C/3A, respectively. By this method, the pipet tip does not come into contact with any tissue surface, thereby precluding inadvertent abrasion of the urethral epithelium. Additional details for performing urethral SHIV challenge using the ‘no contact’ technique are available online: http://dx.doi.org/10.17504/protocols.io.na8dahw.

**Fig 3:**
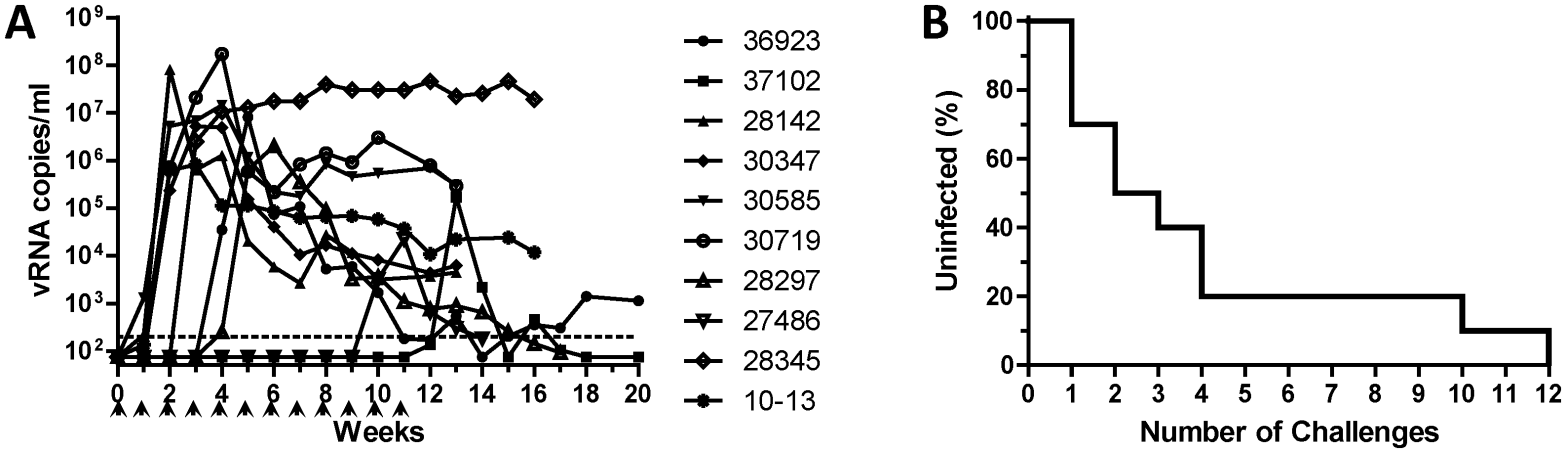
Infection of rhesus macaques following repeated SHIV challenges via combined prepuce pouch and urethral routes. Ten rhesus macaques were challenged once weekly with 200 TCID_50_ SHIV into the prepuce pouch and 16 TCID_50_ SHIV into the urethra using the ‘no-contact’ method, as described, and monitored for the development of positive SHIV viremia by RT-qPCR viral load assay. Viral RNA copies per ml plasma (A) and number of SHIV challenges required to establish systemic infection (B) are presented as in Figs 1 and 2.

### Viral load assay

SHIV RNA in plasma was quantified via real-time reverse transcription PCR assay with a 200 vRNA copies per ml limit of quantitation, as previously described [17]. Statistical comparisons using the log-rank test were performed using Prism-5 software (GraphPad Software, Inc).

## Results

### SHIV acquisition following repeated single-route virus challenges via the prepuce pouch or urethra

To evaluate the inner foreskin/glans or urethra as susceptible sites of penile SHIV acquisition in macaques, groups of 2–3 animals were challenged with SHIV either twice weekly via the prepuce pouch (200 or 600 TCID_50_) or once weekly via the urethra (4 or 16 TCID_50_) and monitored for the development of systemic SHIV infection via positive plasma viral load assay. The number of SHIV challenges required to infect animals in each group was determined from the corresponding plasma viral load kinetics, assuming a minimum 7-day eclipse period as described for SIVmac251 [11].

To identify optimal virus doses for repeat-exposure modeling, we evaluated two different doses for each challenge route (prepuce pouch, urethra). Macaques challenged repeatedly via the prepuce pouch with 200 TCID_50_ SHIV all became infected following a median of 6 challenges (range 3–10, Fig 1A and 1B). In contrast, macaques challenged repeatedly via the prepuce pouch with a relatively higher dose (600 TCID_50_) of SHIV were infected more rapidly (median of 2 challenges, Fig 1C and 1D) (P = 0.046). As a result, the lower dose of 200 TCID_50_ was selected for subsequent studies of SHIV challenge via the prepuce pouch. Similarly, two different doses of SHIV were evaluated for their ability to establish infection following repeated exposures via the urethra. We found only 33% (1/3) of the animals became infected following administration of up to 12 urethral challenges with 4 TCID_50_ SHIV (Fig 2A and 2B). In contrast, three macaques repeatedly challenged via the urethra with 16 TCID_50_ SHIV all became infected following a median of 2 virus exposures (range 2–3, Fig 2C and 2D), which was a significantly fewer number of challenges as compared to the 4 TCID_50_ urethral group (P = 0.022). Thus, a dose of 16 TCID_50_ SHIV was selected for inclusion in subsequent studies of repeated challenges via the urethra.

### SHIV acquisition following repeated simultaneous challenges via prepuce pouch and urethral routes

Although we found that macaques could be infected successfully by either route alone, uncircumcised men are likely to acquire HIV through exposure to multiple tissues including urethra, glans and other penile tissues. Thus, we evaluated the utility of simultaneous dual-route SHIV challenge of the penis. Ten macaques were exposed once weekly to both 200 TCID_50_ SHIV into the prepuce pouch and 16 TCID_50_ into the urethra using the no-contact method. Dual-route challenges resulted in systemic SHIV infection in 100% (10/10) macaques following a median of 2.5 challenges (range of 1–12, Fig 3A and 3B). No significant difference was observed between the number of challenges required to infect macaques following repeated dual-route challenges (Fig 3B), as compared to those administered single-route challenges via either the prepuce pouch (200 TCID_50_, Fig 1B) or urethra (16 TCID_50_, Fig 2D) with a corresponding challenge virus dose.

## Discussion

Penile acquisition of HIV infection contributes substantially to the global HIV epidemic. Notably, the probability for men acquiring HIV following insertive sex (vaginal or rectal) is similar to that for women following receptive vaginal intercourse (risk per 10,000 exposures to an infected source: 4-insertive vaginal, 11-insertive rectal, and 8-receptive vaginal) [18]. Nevertheless, HIV infection via the penis remains relatively understudied, as compared to vaginal or rectal infection routes. As a result, both our mechanistic understanding of how HIV infects through the penis and the development of preventions, such as PrEP, specifically to prevent penile HIV infection has lagged those targeting other routes of HIV transmission.

Macaque models of PrEP efficacy against rectal or vaginal SHIV infection have provided valuable proof-of-concept data to facilitate translation of promising prevention candidates, including oral Truvada^®^ –the first medication approved by the FDA to reduce the risk of sexually acquired HIV infection [3]. Given the significance of penile HIV acquisition globally and the lack of a preclinical penile SHIV acquisition model, we sought to develop an analogous repeat SHIV challenge model of penile infection in rhesus macaques to better understand virus interactions with the penile mucosa and to facilitate translation of penis-specific PrEP modalities towards reducing HIV infection rates in men.

For early infection events of the penis, the relative contributions of different penile tissues and underlying sets of potential target cells remain to be fully determined [19]. The importance of the foreskin to HIV acquisition risk has been demonstrated epidemiologically, with its removal via circumcision resulting in a 50–60% risk reduction [20–22]. While impressive, the fact that circumcision provides only partial protection against penile acquisition of HIV implies that penile tissues other than the foreskin are relevant to acquisition. Anderson, et al hypothesized that the urethra may be the primary site of HIV acquisition in circumcised men, and possibly also in uncircumcised men, as evidenced by the high incidence of sexually transmitted infections at this site (including *N*. *gonorrhoeae* and *T*. *pallidum*, which are associated with increased risk of HIV infection) as well as the urethra’s superficial distribution of HIV target cells [19]. HIV target cells, including Langerhans cells, dendritic cells, CD4+ T cells and macrophages, exhibit wide distributions throughout penile epithelia [23] and explanted (human) foreskin, glans and urethral tissues are susceptible to productive infection with CCR5-utilizing HIV *ex vivo* [12, 24]. Microscopic visualization of fluorescently tagged HIV virions revealed virus penetration into the foreskin, as well as glans tissue, following either *ex vivo* treatment of cadaveric explants or *in vivo* administration of virus to rhesus macaques [25]. We, therefore, incorporated multiple anatomic sites for virus exposure in our penile SHIV challenge model, incorporating exposure to urethra and the tissues around the glans.

We found that macaques could be infected readily with SHIV following repeated challenges administered to the prepuce pouch, or into the urethra using a novel ‘no contact’ technique that avoids compromising the integrity of the urethral epithelium. Within each challenge route, animals exposed to relatively higher doses of SHIV became infected following fewer virus challenges (Figs 1D vs 1B and 2D vs 2B), as expected. Thus, statistically significant dose-response relationships were observed for each single challenge route. In contrast, no significant differences in the numbers of challenges to infection were observed between the dual-route group with either individual single-route component. With the caveat that our single-route group sizes were small (N = 2–3), we observed that the success of infection following repeated challenges with only 16 TCID_50_ SHIV into the urethra (Fig 2D) was similar to that following 600 TCID_50_ SHIV challenges into the prepuce pouch (Fig 1D), suggesting that the urethra is more susceptible to SHIV acquisition than is inner foreskin/glans tissues. However, additional macaque studies will be required, to compare infection outcomes following SHIV challenges of equivalent virus doses delivered via these different routes, to determine whether penile susceptibility truly varies in a site-specific manner. This could be relevant to evaluating HIV prevention for circumcised versus non-circumcised men. Also, the doses for SHIV challenges used here to infect macaques via the prepuce pouch or urethra appear to be lower (by 1–2 logs) than those required for penile infection with SIVmac251 [10]. Determining whether this reflects differences between HIV and SIV envelope interactions with the penile mucosa, differences in inoculation routes or techniques, or other stock-specific variables, will require additional investigation. Nevertheless, our dual-route penile model shows a median of 2.5 challenges to infection, which is similar to that of repeat-exposure rectal or vaginal SHIV challenge models, which are sensitive and have good statistical power for detecting PrEP efficacy [26, 27].

While the relative importance of infected donor mononuclear cells for initiating HIV infections via genital or rectal mucosal routes remains ambiguous [28], studies of polarized penile urethral explant cultures showed cell-associated HIV to be translocated at relatively higher levels than cell-free virus [12]. However, our *in vivo* results clearly demonstrate that inoculation of cell-free virus non-traumatically into the urethra was sufficient to infect macaques readily with SHIV.

In conclusion, we have developed the first repeat-exposure SHIV challenge model of penile HIV acquisition in rhesus macaques, which, uniquely, incorporates concurrent virus exposure to foreskin, glans and urethral tissues. This nonhuman primate model is being used to evaluate biomedical preventions against penile infection, which complement preclinical efficacy data from existing rectal and vaginal challenge models. Additionally, the model may serve as a base for development of more epidemiologically relevant models for HIV prevention studies, using SHIVs containing envelopes from non-clade B or transmitted/founder viruses, or using macaques with penile sexually transmitted co-infections, as we have done for vaginal and rectal infection routes [29, 30].
